# Development and Application of Intragenic Markers for 14 Nitrogen-Use Efficiency Genes in Rice (*Oryza sativa* L.)

**DOI:** 10.3389/fpls.2022.891860

**Published:** 2022-05-09

**Authors:** Pingbo Li, Zhen Li, Xu Liu, Hua Zhang, Qingguo Wang, Nana Li, Hanfeng Ding, Fangyin Yao

**Affiliations:** ^1^Institute of Wetland Agriculture and Ecology, Shandong Academy of Agricultural Sciences, Jinan, China; ^2^Institute of Crop Germplasm Resources, Shandong Academy of Agricultural Sciences, Jinan, China

**Keywords:** rice, nitrogen-use efficiency, haplotype analysis, intragenic marker, germplasm accession

## Abstract

Asian cultivated rice consists of two main subspecies, *xian*/*indica* (XI) and *geng*/*japonica* (GJ), and GJ accessions have significantly lower nitrogen-use efficiency (NUE) than XI accessions. In order to facilitate genetic improvement of NUE in GJ accessions, we conducted haplotype analysis of 14 cloned NUE genes using 36 rice germplasm accessions with high-quality reference genome and developed 18 intragenic markers for elite haplotypes, which were then used to evaluate NUE genes in another 41 genetically diverse germplasm accessions from 12 countries and 71 approved GJ cultivars from northern provinces of China. Our results show that elite haplotypes of 12 NUE genes are mainly existed in XI accessions, but few is distributed in GJ accessions. The number of elite haplotypes carried by an XI accession can reach 10, while that carried by a GJ accession is less than 3. Surprisingly, the elite haplotype of gene *DEP1* is nearly fixed in approved GJ cultivars, and elite haplotypes of gene *MYB61* and *NGR5* have been introduced into some approved GJ cultivars. The developed intragenic markers for NUE genes and evaluated 77 genetically diverse rice accessions could be of great use in the improvement of NUE in GJ cultivars.

## Introduction

Rice is a staple food that feeds more than half of the world population. Nitrogen is a macronutrient that is essential to the growth and grain yield of rice plants. The adoption of semidwarf rice varieties and nitrogen fertilizer has strikingly boosted rice yield since 1960s (Khush, 1999). However, the excess input of nitrogen fertilizer not only contributes little to the increase in grain yield, but also brings great threat to environment in recent years. Therefore, adoption of rice cultivars with high nitrogen-use efficiency (NUE) and reduction of nitrogen input is a promising way to achieve sustainable agriculture. Asian cultivated rice consists of two main subspecies, *xian*/*indica* (XI) and *geng*/*japonica* (GJ), which display significant difference in various traits, including NUE (Hu et al., 2015; Gao et al., 2019). GJ cultivars, favored by consumers in Japan, South Korea, and the northern provinces of China, have low NUE, and massive input of nitrogen fertilizer is the way selected by farmers to improve rice yield. Therefore, genetic improvement of NUE in GJ cultivars is of great necessity to achieve sustainable agriculture. NUE involves nitrogen uptake, translocation, and assimilation, and results in changes in plant height, tiller number, and grain yield in rice (Li et al., 2017). Thus, it is a complex trait controlled by quantitative trait loci (QTL) (Zhou et al., 2017; Tang et al., 2019; Shen et al., 2021). Up to now, 14 genes conferring natural variation in NUE have been cloned and characterized (Table 1). Introgression of elite alleles or haplotypes of these cloned genes into the background of GJ cultivars could be a promising way to improve NUE of GJ cultivars. Marker-assisted selection has been proved to be an effective method in genetic improvement of rice cultivars, of which the premise is the development of effective molecular markers for target genes (Wang et al., 2017; Jiang et al., 2019; Mao et al., 2021).

**TABLE 1:** Information of 14 cloned NUE genes.

Gene symbol	LOC number	Protein encoding^a^	Subcellular localization	Protein function	Elite allele or haplotype	Reference
*OsNPF6.1*	LOC_Os01g01360	A nitrate transporter	Plasma membrane	Uptake and redistribution of nitrate	*OsNPF6.1*^HapB^** that carries two novel binding sites of OsNAC42	Tang et al., 2019
*DNR1*	LOC_Os01g08270	An aminotransferase	Nucleus, cytoplasm	Catalyze the conversion of indole-3-pyruvate to L-Trp, thus antagonizing auxin biosynthesis	Hap.A showing low mRNA abundance	Zhang et al., 2021
*MYB61*	LOC_Os01g18240	A MYB family TF	Nucleus		Hap1 that carries the deletion of a helitron element in the 5′UTR and thus facilitates the binding of NGR2/OsGRF4	Gao et al., 2020
*SBM1*	LOC_Os01g65120	An oligopeptide transporter	Plasma membrane	Promote nitrogen uptake and transport	HapB carried by accession Kasalath and PA64S	Xu et al., 2021
*NGR2*	LOC_Os02g47280	A GRF family TF	Nucleus	Transcriptional activation of nitrogen metabolism related genes	Hap.B or *ngr2* that is carried by accession NM73 and shows high mRNA abundance	Li et al., 2018
*OsNR2*	LOC_Os02g53130	A NADH/NADPH-dependent nitrate reductase		Promote nitrate uptake and utilization	Allele that carries a SNP conferring the Trp_779_ to Arg_783_ substitution in the NAD(P) binding domain	Gao et al., 2019
*NGR5*	LOC_Os05g32270	An AP2-domain TF	Nucleus	Facilitate nitrogen-dependent recruitment of polycomb repressive complex 2 to repress branching-inhibitory genes *via* H3K27me3 modification	Hap.2 that is carried by accession Guichao2 and shows high mRNA abundance	Wu et al., 2020
*OsTCP19*	LOC_Os06g12230	A TCP family TF	Nucleus	Transcriptional repression of DLT genes	*OsTCP19*-H that carries a 29-bp deletion in the promoter and thus enhances the *trans*-repression of LBD proteins	Liu et al., 2021
*ARE1*	LOC_Os08g12780	A protein with unknown function	Chloroplast		*ARE1*^9311^ and *ARE1^*MH*63^* that carry small insertions in the promoter and show low mRNA abundance	Wang W. et al., 2018
*DEP1*	LOC_Os09g26999	An atypical γ subunit of G proteins	Nucleus		*dep1* that is carried by accession Ballila and carries the replacement of a 637-bp stretch of the middle of exon 5 by a 12-bp sequence	Huang et al., 2009; Sun et al., 2014
*OsNAC42*	LOC_Os09g32040	A NAC family TF	Nucleus	Transcriptional activation of *OsNPF6.1*	*OsNAC42*^HapC^** that is carried by accession IR36 and shows high mRNA abundance	Tang et al., 2019
*OsNLP4*	LOC_Os09g37710	An NLP family TF	Nucleus	Transcriptional activation of *OsNiR*	*OsNLP4^HapB^* that shows high mRNA abundance	Yu et al., 2021
*NRT1.1B*	LOC_Os10g40600	A nitrate transporter	Plasma membrane	Uptake and transport of nitrate	Allele that carries a SNP conferring the Thr_327_ to Met_327_ substitution in the central cytoplasmic loop	Hu et al., 2015
*TOND1*	LOC_Os12g43440	A thaumatin protein	Plasma membrane		H1 carried by accession Teqing and 9311	Zhang et al., 2015

*^a^TF, transcription factor.*

### Objective

Our aim was to develop effective intragenic markers for the 14 cloned NUE genes in rice, and then to evaluate NUE genes of some germplasm accessions and approved GJ cultivars, which could guide the following genetic improvement. Haplotype analysis of the 14 NUE genes from 36 rice accessions with high-quality reference genome provided many useful sequence variations for marker development, and developed intragenic markers facilitated the evaluation of NUE genes in germplasm accessions and approved cultivars.

## Materials and Methods

### Rice Accessions and Cultivars

Three panels of rice materials were exploited in this study. The first panel consisted of 36 rice accessions with reference genome (Table 2), including GJ accession Nipponbare (NIP) (International Rice Genome Sequencing Project, 2005), 31 genetically diverse rice accessions (Qin et al., 2021), XI accessions Huazhan and Tianfeng (Zhang et al., 2022), and XI accessions Minghui63 and Zhenshan97 (Song et al., 2021). The second panel consisted of 41 genetically diverse rice accessions from 12 countries (Supplementary Table S16). The third panel consisted of 71 GJ cultivars approved in the northern provinces of China, including 2 from Anhui, 1 from Henan, 9 from Jiangsu, 39 from Shandong, 1 from Tianjin, and 19 approved in several provinces (Supplementary Table S17). The subpopulation of accessions in the first panel and second panel referred to a previous study (Wang W. et al., 2018).

**TABLE 2:** Haplotypes of 14 NUE genes in the 36 rice accessions with reference genome.

Accessions	Orign^a^	Subpopu.^b^	NUE genes*^c^*														No. of elite haplotypes
			*OsNPF6.1*	*DNR1*	*MYB61*	*SBM1*	*NGR2*	*OsNR2*	*NGR5*	*OsTCP19*	*ARE1*	*DEP1*	*NAC42*	*OsNLP4*	*NRT1.1B*	*TOND1*	
N22	India	cA	HapD	n.d.	n.d.	**HapB**	HapE	**HapB**	HapD	**HapB**	n.d.	HapF	HapD	**HapB**	n.d.	n.d.	4
Basmati 1	Pakistan	cB	HapA	HapC	**HapB**	HapA	HapE	n.d.	HapA	n.d.	n.d.	HapA	HapE	HapA	HapA	n.d.	1
LJ	YN, China	GJ-adm	HapA	HapD	HapC	HapA	HapE	HapA	HapA	HapA	HapA	HapA	HapA	HapA	HapA	HapC	0
NamRoo	Thailand	GJ-sbtrp	HapA	HapA	HapC	HapA	HapE	HapA	HapA	HapA	n.d.	HapA	HapA	HapA	HapA	HapC	0
2428	JS, China	GJ-tmp	HapA	HapD	HapC	HapA	HapC	HapA	HapA	HapA	HapA	HapA	HapA	HapA	HapA	HapC	0
DHX2	HLJ, China	GJ-tmp	HapA	HapA	HapC	HapA	HapE	HapA	HapA	HapA	HapA	HapA	HapA	HapA	HapA	HapC	0
Kosh	Japan	GJ-tmp	HapA	HapA	HapA	HapA	HapC	HapA	HapA	HapA	HapA	HapA	HapA	HapA	HapA	HapC	0
KY131	Japan	GJ-tmp	HapA	HapA	HapC	HapA	HapC	HapA	HapA	HapA	HapA	HapA	HapA	HapA	HapA	HapC	0
NIP	Japan	GJ-tmp	HapA	HapA	HapA	HapA	HapC	HapA	HapA	HapA	HapA	HapA	HapA	HapA	HapA	HapA	0
ZH11	TJ, China	GJ-tmp	HapA	HapA	HapC	HapA	HapC	HapA	HapA	HapA	HapA	HapA	HapA	HapA	HapA	HapC	0
Lemont	United States	GJ-trp	HapA	HapD	HapC	HapD	HapE	HapA	HapA	HapA	HapA	HapA	HapA	HapA	HapA	HapC	0
HZ	China	XI	HapA	**HapB**	**HapB**	HapD	HapA	**HapB**	HapD	**HapB**	**HapC**	HapC	HapC	**HapB**	**HapB**	**HapB**	7
TFB	GD, China	XI	**HapB**	**HapB**	**HapB**	HapC	**HapB**	**HapB**	HapD	HapA	**HapC**	HapF	HapA	**HapB**	**HapB**	HapC	8
CN1	SC, China	XI-1A	**HapB**	**HapB**	**HapB**	HapC	HapD	**HapB**	HapE	HapA	**HapC**	HapF	HapE	**HapB**	**HapB**	**HapB**	8
D62	SC, China	XI-1A	**HapB**	**HapB**	**HapB**	HapD	HapC	**HapB**	HapD	HapA	**HapC**	HapF	**HapB**	**HapB**	**HapB**	**HapB**	9
DG	FJ, China	XI-1A	HapC	**HapB**	**HapB**	HapC	HapD	**HapB**	HapD	HapA	**HapC**	HapD	HapD	**HapB**	**HapB**	HapC	6
FS32	n.d.	XI-1A	HapC	**HapB**	**HapB**	n.d.	HapA	**HapB**	HapE	HapA	**HapC**	HapD	n.d.	**HapB**	**HapB**	**HapB**	7
G46	SC, China	XI-1A	**HapB**	**HapB**	**HapB**	n.d.	HapA	**HapB**	HapE	HapA	**HapC**	HapF	HapE	**HapB**	**HapB**	HapC	7
II32	HN, China	XI-1A	**HapB**	**HapB**	**HapB**	HapC	**HapB**	**HapB**	HapE	HapA	**HapC**	HapD	HapA	**HapB**	**HapB**	HapC	8
ZS97	ZJ, China	XI-1A	**HapB**	**HapB**	**HapB**	HapC	**HapB**	**HapB**	HapE	HapA	**HapC**	HapD	HapA	**HapB**	**HapB**	HapC	8
9311	JS, China	XI-1B	HapA	**HapB**	**HapB**	HapC	HapA	**HapB**	HapE	HapA	**HapC**	HapD	HapD	**HapB**	**HapB**	**HapB**	6
G8	GD, China	XI-1B	HapA	**HapB**	**HapB**	HapC	**HapB**	**HapB**	HapE	HapA	**HapC**	HapC	HapD	**HapB**	**HapB**	**HapB**	8
IR64	IRRI	XI-1B	HapA	**HapB**	**HapB**	HapD	**HapB**	**HapB**	HapC	**HapB**	**HapB**	HapE	**HapB**	**HapB**	**HapB**	**HapB**	10
J4115	HN, China	XI-1B	HapA	**HapB**	**HapB**	HapD	HapE	**HapB**	**HapB**	HapA	**HapB**	HapC	**HapB**	**HapB**	**HapB**	**HapB**	9
R498	SC, China	XI-1B	HapC	**HapB**	**HapB**	HapD	HapA	**HapB**	**HapB**	HapA	**HapB**	HapC	**HapB**	**HapB**	**HapB**	**HapB**	9
R527	JX China	XI-1B	HapA	**HapB**	**HapB**	HapD	**HapB**	**HapB**	**HapB**	HapA	**HapB**	HapE	**HapB**	**HapB**	**HapB**	**HapB**	10
S548	SC, China	XI-1B	HapA	**HapB**	**HapB**	HapD	HapA	HapA	**HapB**	**HapB**	**HapB**	HapC	HapC	**HapB**	**HapB**	**HapB**	8
Y3551	SC, China	XI-1B	HapA	**HapB**	**HapB**	HapD	HapA	**HapB**	**HapB**	HapA	**HapC**	HapC	HapE	**HapB**	**HapB**	**HapB**	8
Y58S	HN, China	XI-1B	HapA	**HapB**	**HapB**	HapD	HapE	**HapB**	HapA	HapA	**HapC**	HapC	**HapB**	**HapB**	**HapB**	**HapB**	7
TM	Madagascar	XI-2	HapA	**HapB**	n.d.	HapC	HapE	**HapB**	HapD	**HapB**	n.d.	HapD	HapD	**HapB**	**HapB**	**HapB**	6
TUMBA	Indonesia	XI-3	HapA	**HapB**	**HapB**	HapC	HapD	**HapB**	HapD	**HapB**	n.d.	HapE	**HapB**	**HapB**	**HapB**	**HapB**	8
FH838	SC, China	XI-adm	HapA	**HapB**	**HapB**	HapD	HapA	**HapB**	**HapB**	HapA	**HapB**	HapE	**HapB**	**HapB**	**HapB**	HapC	8
G630	Guyana	XI-adm	HapA	**HapB**	**HapB**	HapD	HapA	**HapB**	**HapB**	HapA	n.d.	HapC	HapE	**HapB**	**HapB**	HapC	6
MH63	FJ, China	XI-adm	HapA	**HapB**	**HapB**	HapD	HapA	**HapB**	**HapB**	HapA	**HapB**	HapC	HapE	**HapB**	**HapB**	**HapB**	8
WSSM	GD, China	XI-adm	HapA	**HapB**	**HapB**	HapD	HapA	**HapB**	HapD	HapA	**HapC**	HapE	**HapB**	**HapB**	**HapB**	n.d.	7
YX1	SC, China	XI-adm	**HapB**	**HapB**	**HapB**	HapC	**HapB**	**HapB**	**HapB**	HapA	**HapC**	HapD	HapA	**HapB**	**HapB**	**HapB**	10

*^a^FJ, Fujian; GD, Guangdong; HLJ, Heilongjiang; HN, Hunan; JS, Jiangsu; JX, Jiangxi; SC, Sichuan; TJ, Tianjin; YN, Yunnan; ZJ, Zhejiang. ^b^The subpopulation information referred to the paper reported by Qin et al. (2021). ^c^n.d., not determined. If the genomic sequence of a gene from an accession could not be extracted, or carried many novel variations, the haplotype of the gene would be termed as n.d.*

### Haplotype Analysis

The genomic sequences of 14 cloned NUE genes from 36 rice accessions with reference genome were extracted using the software BioEdit (Hall, 1999) and software SeqBuilder from the Lasergene package (DNASTAR, lnc., Madison, USA), including 2-kb region upstream of the start codon termed as 5′ untranslated region (5′UTR), the coding region, and 1-kb region downstream of the stop codon termed as 3′UTR. Elite alleles of *NGR2*, *NGR5*, *DEP1*, and *IR36* were reported to be carried by a very few accessions not included in the 36 accessions, and their genomic sequences could not be found in related databases and papers. Therefore, genomic sequences of allele *ngr2* from accession NM73, allele *NGR5* from accession Guichao2, allele *dep1* from accession Ballila, and allele *NAC42* from accession IR36 were resequenced using the Sanger sequencing method and assembled using the software SeqMan from the Lasergene package, and related primers were listed in Supplementary Table S1. Sequence alignment for each gene was conducted using the software MEGA 7. Haplotype analysis of each gene was conducted, based on a combination of functional variations reported in previous studies and sequence variations identified in this study. For each NUE gene, the haplotype of accession NIP was defined as HapA, and reported elite haplotype was defined as HapB (Table 2 and Supplementary Tables S2–S15).

### Marker Development

Several rules were followed during marker development. First of all, target variations should be unique to the elite haplotype of each gene. Secondly, an insertion/deletion (Indel) whose size was between 5 and 30 bp was given priority, and the Indel marker with amplification fragment between 100 and 300 bp was developed. Thirdly, if no Indel could be selected, a single-nucleotide polymorphism (SNP) that could be transformed into a common restriction enzyme site was taken into consideration, and then the cleaved amplified polymorphic sequences (CAPS) marker with amplification fragment between 100 and 500 bp or derived CAPS (dCAPS) marker with amplification fragment between 100 and 300 bp was developed. Finally, if no variation could meet the second and third rules, a penta-primer amplification-refractory mutation system (PARMS) marker was designed (Qing et al., 2018).

Indel markers were developed using the software Primer Premier 6 (PREMIER Biosoft, San Francisco, USA). CAPS markers and dCAPS markers were developed using the website dCAPS Finder 2.0^1^ (Neff et al., 2002) and the software Primer Premier 6. PARMS markers were developed using the website SNPWay^2^.

### Marker Analysis

For Indel markers, CAPS markers and dCAPS markers, the PCR reaction was 3-μl genomic DNA with a concentration of 20 ng/μl, 0.5 μl of each primer with a concentration of 10 μM/L, 10-μl 2*Taq PCR MasterMix, and 6 μl of ddH_2_O. The PCR profile was 3 min at 94°C for denaturation, followed by 32 cycles of 94°C for 30 s, 55°C for 30 s, and 72°C for 1 min/kb, then by 3 min at 72°C for extension, and finally by 1 min at room temperature. The PCR products of Indel markers were run on 4% polyacrylamide gels with a voltage of 160 V for 80 min, and bands were then revealed using a silver staining procedure: gels were washed for 1 min with dH_2_O, followed by incubated for 10 min in staining solution (0.4 g AgNO_3_, 400 ml dH_2_O) with gentle shaking, then washed two times for 1min with dH_2_O, then incubated for 4 min in developing solution (6 g NaOH, 2 ml CH_3_CHO, 400 ml dH_2_O) with gentle shaking, and finally washed for 1 min with water. The PCR products of dCAPS markers and CAPS markers were digested with corresponding restriction enzymes purchased from Takara Biomedical Technology Co., LTD., Beijing, China, according to the recommended reaction and temperature. The digested products of dCAPS markers were analyzed as that of Indel markers described above. The digested products of CAPS markers were run 2% agarose gels containing GeneRed nuclelic acid dye with a voltage of 120 V for 20 min, and bands were developed in UVP EC3 Imaging System (Analytik Jena AG, Jena, Germany).

For PARMS markers, the PCR reaction was 2-μl genomic DNA with a concentration of 20 ng/μl, 0.15 μl of each forward primer and 0.4 μl of common reverse primer with a concentration of 10 μM/L, 5-μl 2*PARMS master mix containing two universal fluorescent primers, and 2.3 μl of ddH_2_O. The PCR profile was 15 min at 94°C for denaturation, followed by 10 cycles of 94°C for 20 s and 65°C (with each cycle minus 0.8°C) for 1 min, then by 28 cycles of 94°C for 20 s and 57°C for 1 min, and finally by 1 min at temperature. The PCR products were analyzed using ABI QuantStudio 5 fluorescent quantitative PCR system (Applied Biosystem, Foster, USA).

## Results

### Haplotype Analysis of 14 Nitrogen-Use Efficiency Genes

In order to have a closer examination of sequence variations in the 14 cloned NUE genes (Table 1), we extracted the genomic sequences from the 36 rice accessions with reference genome, including 2-kb 5′UTR, the coding region, and 1-kb 3′UTR. We also sequenced the genomic sequence of allele *ngr2* from accession NM73, allele *NGR5* from accession Guichao2, allele *dep1* from accession Ballila, and allele *NAC42* from accession IR36, which were reported elite alleles and whose sequences were not accessible. Subsequently, we performed sequence alignment and haplotype analysis of each gene, with the type of accession NIP termed as HapA and reported elite haplotype termed as HapB (Table 2).

For *OsNPF6.1*, four haplotypes were identified, including the two reported by Tang et al. (2019; Table 2 and Supplementary Table S2). Compared to HapA, both HapB and HapC carried two SNPs at positions −1041 and −443 that created two new binding sites for protein OsNAC42. In addition, HapB carried a non-synonymous SNP at position + 125 and HapC carried two non-synonymous SNPs at positions + 506 and + 535 in the first exon.

For *DNR1*, four haplotypes were identified, including the three types reported by Zhang et al. (2021), which were HapA (Hap.B-TEJ), HapB (Hap.A), HapC, and HapD (Hap.B-TRJ) (Table 2 and Supplementary Table S3). The four types were distributed in GJ-tmp, XI, cA/cB, and GJ-trp accessions, respectively (Table 2). Compared to HapA, HapB carried a 520-bp deletion, a 2-bp deletion and twenty SNPs in 5′UTR, five Indels and eighteen SNPs in the coding region, and three SNPs in the 3′UTR. In addition, both HapC and HapD carried the 520-bp deletion at position -1201.

For *MYB61*, three haplotypes were identified, in agreement with the three types reported by Gao et al. (2020), which were HapA (Hap3), HapB (Hap1), and HapC (Hap2) (Table 2 and Supplementary Table S4). HapC differed from HapA by an SNP G/A at position −323, of which both were distributed in GJ accessions. HapB, the type distributed in XI accessions, carried the deletion of 975-bp helitron at position −446 that was close to the binding site of NGR2/OsGRF4 in the 5′UTR, and a non-synonymous SNP at position + 1224 in the coding region relative to the other two types.

For *SBM1*, four haplotypes were identified, including the three types reported by Xu et al. (2021; Table 2 and Supplementary Table S5). HapA and HapB were distributed mainly in GJ and cA accessions, respectively, while both HapC and HapD were distributed mainly in XI accessions. Compared to HapA and HapC, HapB carried two Indels and fifteen SNPs in the 5′UTR, a 3-bp deletion and two SNPs in the coding region, and three SNPs in the 3′UTR. HapD carries a 1,204-bp deletion in the 5′UTR and a non-synonymous SNP at position + 2 which disrupted the translation start site, relative to the other three types (Supplementary Figure S1).

For *NGR2*, five haplotypes were identified, including the three reported by Li et al. (2018) (Table 2 and Supplementary Table S6). HapB carried 14 SNPs at positions −1806, −1754, −1738, −1691, −1314, −935, −878, −844, −795, +511, +641, +654, +2275, and +3330 relative to the other four types, and was distributed in XI accessions. In addition, the two rare SNPs (+1187T > A and +1188C > A) preventing miR396-meidated cleavage of mRNA were not carried by all the 36 accessions.

For *OsNR2*, two haplotypes were identified, based on the functional SNP at position + 2692 reported by Gao et al. (2019; Table 2 and Supplementary Table S7). HapA and HapB were distributed in GJ and XI accessions, respectively. HapB differed from HapA by a 26-bp insertion and nine SNPs in the 5′UTR, a 12-bp insertion and three non-synonymous SNPs in the coding region, and four SNPs in the 3′UTR.

For *NGR5*, five haplotypes were identified, in agreement with the five reported by Wu et al. (2020), which were HapA (Hap1), HapB (Hap2), HapC (Hap3), HapD (Hap4), and HapE (Hap5) (Table 2 and Supplementary Table S8). Among those, HapB, HapC, and HapD shared many variations relative to the other two types, and differentiated from each other by an SNP, respectively. HapB carried an SNP at position + 3326 in the sixth intron, and HapC carried an SNP at position + 4020 in the eighth exon.

For *OsTCP19*, two haplotypes were identified, based on the functional 29-bp deletion at position −2022 reported by Liu et al. (2021; Table 2 and Supplementary Table S9).

For *ARE1*, three haplotypes were identified, in agreement with the three reported by Wang et al. (2018), which were HapA (*ARE1^NPB^*), HapB (*ARE1^*MH*63^*), and HapC (*ARE1*^9311^) (Table 2 and Supplementary Table S10). Compared to HapA, HapB carried a 6-bp insertion and 10 SNPs in the 5′UTR, 11 SNPs in the coding region, and a 6-bp insertion and three SNPs in the 3′UTR. HapC carried a 2-bp deletion, a 7,808-bp insertion and eight SNPs in the 5′UTR, ten SNPs in the coding region, and a 6-bp insertion in the 3′UTR.

For *DEP1*, six haplotypes were identified (Table 2 and Supplementary Table S11). The functional variation – the replacement of a 637-bp segment of the middle of exon 5 by a 12-bp sequence, is carried by accession Ballila but not by all 36 accessions (Huang et al., 2009).

For *OsNAC42*, five haplotypes were identified (Table 2 and Supplementary Table S12). Compared to HapA, HapB carried eight SNPs in the 5′UTR, eleven SNPs in the coding region, and a 27-bp deletion and six SNPs in the 3′UTR.

For *OsNLP4*, two haplotypes were identified, in agreement with the two reported by Yu et al. (2021), which were distributed in GJ and XI accessions, respectively (Table 2 and Supplementary Table S13). Compared to HapA, HapB carried a 6-bp deletion and eight SNPs in the 5′UTR, five SNPs in the coding region, and five SNPs in the 3′UTR.

For *NRT1.1B*, two haplotypes were identified, based on the functional SNP at position + 3019 reported by Hu et al. (2015), which were distributed in GJ and XI accessions, respectively (Table 2 and Supplementary Table S14). Compared to HapA, HapB carried a 3-bp insertion and thirteen SNPs in the 5′UTR, five Indels and twenty-two SNPs in the coding region, and two Indels and nine SNPs in the 3′UTR.

For *TOND1*, three haplotypes were identified, including the elite type reported by Zhang et al. (2015), which was HapB (H1) (Table 2 and Supplementary Table S15). Compared to HapA and HapC, HapB carried an 8-bp insertion and three SNPs in the 5′UTR, and two non-synonymous SNPs in the coding region.

### Development of Intragenic Markers for Nitrogen-Use Efficiency Genes

**FIGURE 1:**
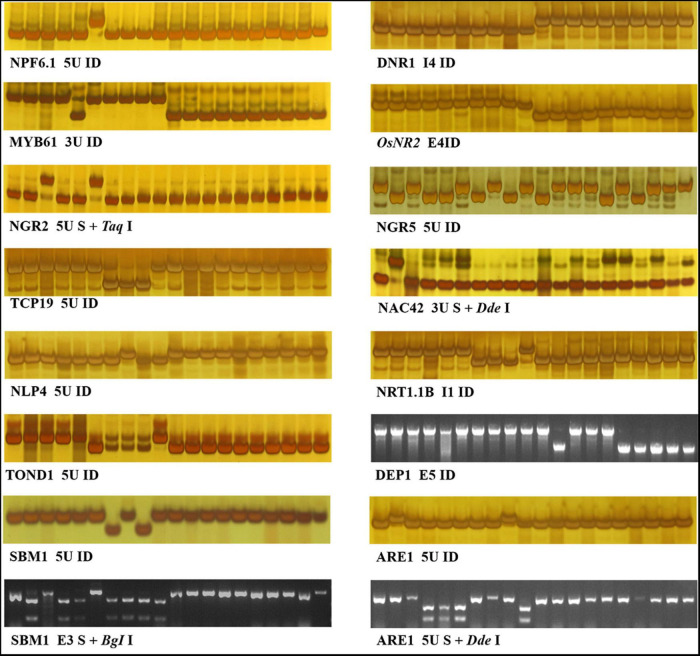
Indel, CAPS, and dCAPS markers for 14 NUE genes. The 20 accessions used for marker evaluation were 9311, IR36, NM73, Guicao 2, Huagengxian 74, ZS97, Kasalath, Basmati 370, Dular, NJ11, 02428, Ballila, NIP, Kosh, KY131, Runnong11, Suxiu 867, Huaidao 5, Xudao 3, and Huageng5 (here from left to right). The first 15 accessions were germplasm accessions, including donors of the 14 NUE genes, and the other 5 accessions were mainstay cultivars in the Huang–Huai rice area of China. In the figure of marker “TOND1 5U ID,” no band was amplified from accession Kasalath, Basmati 370, and Dular.

**FIGURE 2:**
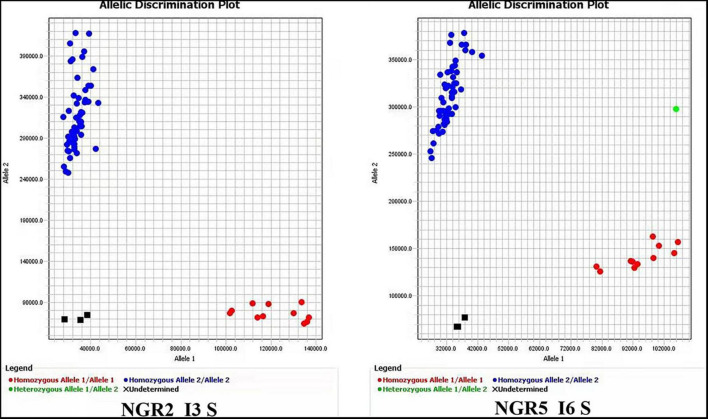
PAMRS markers for *NGR2* and *NGR5.* The accessions used for marker evaluation were the 41 germplasm accessions in Supplementary Table S16 and the first 16 accessions in Figure 1.

In order to develop effective markers for NUE genes, we focused on the variations only carried by HapB of each gene. As shown in Table 3, a total of 18 markers were developed for the 14 genes including twelve Indel markers, three CAPS makers, a dCAPS marker, and two PARMS markers. The effectiveness of 16 markers was evaluated by 20 germplasm accessions (Figure 1), and that of the two PARMS markers was evaluated by 57 germplasm accessions (Figure 2).

**TABLE 3:** Intragenic markers for the 14 NUE genes.

Gene	Variation position^a^	Marker type	Marker name^b^	Primer sequence		Reference size^c^	Restriction enzyme	Target Haplotype
*OsNPF6.1*	–1197	Indel	NPF6.1 5U ID	F	TAATGTCCTTTCCCGTGTT	220 (+20)		HapB
				R	GTACTACTTCGGCTGTCC			
*DNR1*	+2165	Indel	DNR1 I4 ID	F	CGTCAATTATGGTTACCTCTG	171 (–10)		HapB
				R	GCATCTCATAGAACTGAAGAAG			
*MYB61*	+2346	Indel	MYB61 3U ID	F	ACTTGAATACAGGCATGGAA	205 (+26)		HapB
				R	CGTTATGCTTGTTGCTTGA			
*SBM1*	–325	Indel	SBM1 5U ID	F	TCGGGTGTACTACGGATC	90 (–8)		HapB
				R	GCACATACATATCAGGGA			
	+865	CAPS	SBM1 E3 S	F	TCTTCAACTGGATCAACTTC	380/(100,280)	*BgI* I	HapB, HapD
				R	TACATCACGGTGGTCATC			
*NGR2*	–935	CAPS	NGR2 5U S	F	TCATTGACCTACGGTTGC	(249,24)/273	*Taq*I	HapB
				R	GCTGCTCCAACATCTTCT			
	+1701	PARMS	NGR2 I3 S	FC	GAAGGTGACCAAGTTCATGCTAGTAGTACTACTTCGATTTGGTGCT			HapB
				FT	GAAGGTCGGAGTCAACGGATTAGTAGTACTACTTCGATTTGGTGCC			
				R	GCAGTGCAGAGAGTAAAGTTTCAG			
*‘OsNR2*	+2202	Indel	NR2 E4 ID	F	TCACGTCCATCGTTGAGA	120 (+12)		HapB
				R	ACAGGCTCTTCTTGTCCAT			
*NGR5*	–1565	Indel	NGR5 5U ID	F	AGAACACACGGGATAGGAT	210 (–13)		HapB, HapC, HapD
				R	GAATCACTTGCTCGCTAGA			
	+3326	PARMS	NGR5 I6 S	FC	GAAGGTGACCAAGTTCATGCTATCTCGCTGTCCTAAACGACTTCC			HapB
				FT	GAAGGTCGGAGTCAACGGATTATCTCGCTGTCCTAAACGACTTCT			
				R	GAATTACGTACACCCTCCGTACTC			
*OsTCP19*	–2022	Indel	TCP19 5U ID	F	AACTCTTCAGGGTTCTTGC	231 (–29)		HapB
				R	GTGCCGTGTCACATAGAG			
*ARE1*	–393	Indel	ARE1 5U ID	F	CCGTGTCTTATCCACTCC	115 (+6)		HapB
				R	ATGGGGATCGATACGATG			
	–723	CAPS	ARE1 5U S	F	TTAACACTTGTGGCAATGAC	320/(100,210)	*Dde*I	HapC
				R	CTAGTACCGTATTGGCTGTT			
*DEP1*	+3454	Indel	DEP1 E5 ID	F	GACCAAGGTGCCTCAATT	1085 (–615)		HapB
				R	TTCAACCTCGTCTCATAGC			
*NAC42*	+3508	dCAPS	NAC42 3U S	F	TACGTGACTTCGACGGCtGA	(100,20)/120	*Dde*I	HapB
				R	ACGGTCCAAATGCTGCTTCG			
*NLP4*	–1771	Indel	NLP4 5U ID	F	AAGTCCTTCCTAAACTGAGA	136 (–6)		HapB
				R	GGTCTGTTCCAAACAAAGAT			
*NRT1.1B*	+2825	Indel	NRT1.1B I1 ID	F	CATATTTGTTGGCTGCTAAC	178 (+16)		HapB
				R	GGTGGTTCTAACGGTCAA			
*TOND1*	–1205	Indel	TOND1 5U ID	F	TTTGGGTCCCTGACAATA	153 (+8)		HapB
				R	TGGAACAACTCAAGTAGCA			

*^a^Variation position is the position of target variation selected for marker development, relative to the start codon ATG. Target variation and its position were also shown in the supplementary table of each gene.*

*^b^The name of each marker was defined as “gene name + variation position + variation type.” For example, “NPF6.1 5U ID” indicates that the target variation is an Indel in the 5′UTR of NPF6.1, and “SBM1 E3 S” indicates that the target variation is an SNP in the third exon of SBM1.*

*^c^For Indel markers, the number out of bracket is the size of amplification fragment from accession NIP, and the content in bracket is the information of target Indel carried by the elite haplotype. For CAPS and dCAPS markers, the number before “/”is the size of fragment digested with corresponding restriction enzyme from NIP, while the number behind “/”is that from accessions carrying elite haplotype.*

*SBM1* was reported as a negative regulator of NUE and grain yield, and the elite type HapB carried by accession Kasalath was a type with weak function (Xu et al., 2021). HapD carried a 1,204-bp deletion in the 5′UTR region and a non-synonymous SNP at position + 2 that would disrupt the translation start site, and was likely to have a better performance than HapB (Supplementary Table S5). Thus, the CAPS marker “SBM1 E3 S” together with restriction enzyme *BgI* I was developed to select HapB and HapD, which could also differentiate the two types together with the Indel marker “SBM1 5U ID.”

Two elite types of *ARE1* were reported, which were carried by accession Minghui63 and 9311, respectively (Wang et al. 2018). However, the 6-bp insertion reported in the promoter region of accession 9311 was not observed in all the 36 accessions with reference genome, including the resequenced accession 9311. A 7,808-bp insertion in the HapC carried by 15 accessions was observed at position −466, which might reduce the expression of *ARE1* and make it an elite type (Supplementary Table S10). Thus, the CAPS marker “ARE1 5U S” together with restriction enzyme *Dde*I was developed to select HapC.

*NGR5* was classified into five haplotypes, among which HapB, HapC, and HapD differentiated from each other only by one SNP (Supplementary Table S8). HapB was reported to be superior to HapC and HapD, but the SNP in the sixth intron could not account for the difference. We then examined the 7-kb region upstream of the start codon, and found that no additional variation could further differentiate the three types (data not shown). Therefore, it was likely that all the three types were elite, and an Indel marker “NGR5 5U ID” was developed for the 13-bp deletion at position −1565, which was shared by the three types. In addition, a PARMS marker “NGR5 In6 S” was developed to select HapB only.

### Evaluation of Nitrogen-Use Efficiency Genes in Germplasm Accessions and Approved Cultivars

In order to facilitate the genetic improvement of NUE in GJ cultivars, we examined the haplotypes of 14 NUE genes in 41 germplasm accessions from 12 countries (Supplementary Table S16) and 71 GJ cultivars approved in China (Supplementary Table S17) using the 18 makers developed above.

**FIGURE 3:**
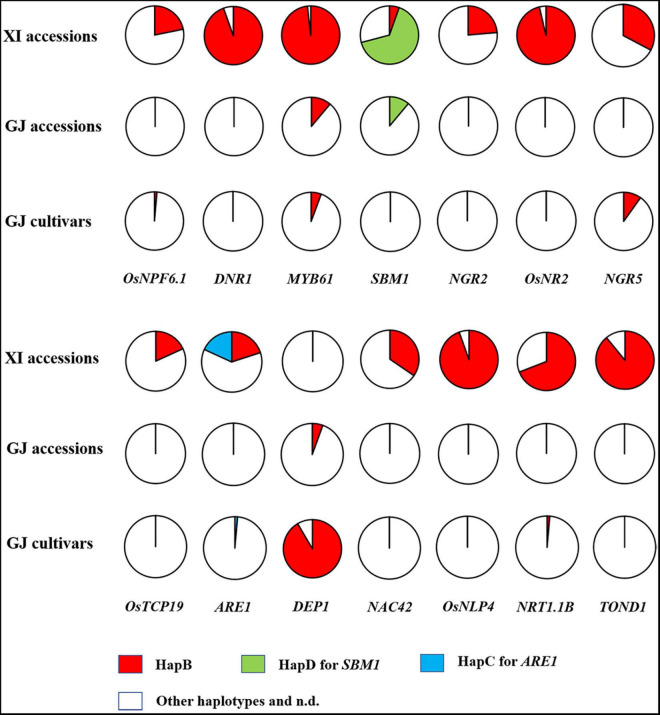
Distribution of elite haplotypes of 14 NUE genes in 55 XI accessions, 18 GJ accessions, and 71 GJ cultivars.

We firstly analyzed the distribution of elite NUE haplotypes in 77 germplasm accessions, including the 36 accessions with reference genome (Table 2) and 41 accessions examined with developed markers (Supplementary Table S16). The elite haplotype of each NUE gene was mainly existed in XI accessions, except for that of *SBM1* and *DEP1* (Figure 3). The number of elite haplotypes carried by an XI accession ranged from 4 to 10, with an average value of 7.56. Three XI accessions, namely, IR64, R527, and YX1, carried 10 elite haplotypes. In contrast, few elite haplotypes were carried by GJ accessions, and 3 accessions carried only one elite haplotype, which were Ballila carrying allele *dep1*, and IRAT261 and Suyunuo carrying *MYB61*.

We subsequently analyzed the distribution of elite NUE haplotypes in GJ cultivars approved in the northern provinces of China. A very few elite haplotypes were carried by these cultivars, and the number of elite haplotypes carried by an accession ranged from 0 to 3. Surprisingly, the elite allele *dep1* was carried by 65 out of 71 accessions (Figure 3 and Supplementary Table S17). The HapB of *NGR5* was carried by six accessions and the HapB of *MYB61* was carried by four accessions. In addition, the HapB of *OsNPF6.1* and *NRT1.1B* was carried by an accession, respectively.

## Discussion

In this study, haplotype analysis of the 14 NUE genes using high-quality genomic sequences facilitated the identification of some important variations and haplotypes. For example, HapC of *ARE1* carried by XI accession 9311 contained several variations in the 5′UTR that was different from reported variations, including a 7,808-bp insertion at position −466 in this study (Supplementary Table S10) and a 6-bp insertion at position −314 in reported study (Wang Q. et al., 2018). HapD of *SBM1* was carried by 14 out of 36 accessions in this study (Supplementary Table S5), but was not identified from 1,140 rice accessions of broad genetic diversity (Xu et al., 2021). Therefore, high-quality genomic sequence was of great importance in haplotype analysis. Intragenic or functional variations that resided in the region covering from 2-kb upstream of start codon to 1-kb downstream of stop codon were selected for marker development. The markers developed in this study were ideal markers for its co-segregating with elite alleles. Haplotype analysis of the 14 NUE genes from 77 genetically diverse rice germplasm accessions revealed that the number of elite haplotypes carried by an XI accession ranged from 4 to 10, while that carried by a GJ accession ranged from 0 to 3, which gives a good explanation of the NUE difference between the two subpopulations of Asian rice cultivars (Table 2 and Supplementary Table S16). Finally, developed intragenic markers for NUE genes and evaluated germplasm accessions in this study could be of great use in guiding the improvement of NUE in GJ cultivars in the future.

## Data Availability Statement

The original contributions presented in the study are included in the article/Supplementary Material, further inquiries can be directed to the corresponding author/s.

## Author Contributions

PL performed most of the experiments and wrote the manuscript. ZL participated in marker development and materials evaluation. XL, HZ, and QW participated in materials evaluation. NL and HD provided some rice accessions and cultivars. FY designed and supervised the study. All authors contributed to the article and approved the submitted version.

## Conflict of Interest

The authors declare that the research was conducted in the absence of any commercial or financial relationships that could be construed as a potential conflict of interest.

## Publisher’s Note

All claims expressed in this article are solely those of the authors and do not necessarily represent those of their affiliated organizations, or those of the publisher, the editors and the reviewers. Any product that may be evaluated in this article, or claim that may be made by its manufacturer, is not guaranteed or endorsed by the publisher.

## Footnotes

1
http://helix.wustl.edu/dcaps/dcaps.html

2
http://www.snpway.com/
