# Supplementary material for: Development and Application of Intragenic Markers for 14 Nitrogen-Use Efficiency Genes in Rice (Oryza sativa L.)
Source: Front Plant Sci. 2022 May 9;13:891860. doi: 10.3389/fpls.2022.891860 (PMC9125075; doi:10.3389/fpls.2022.891860)
Supplement: Supplementary file 4 [file Table_4.DOCX]

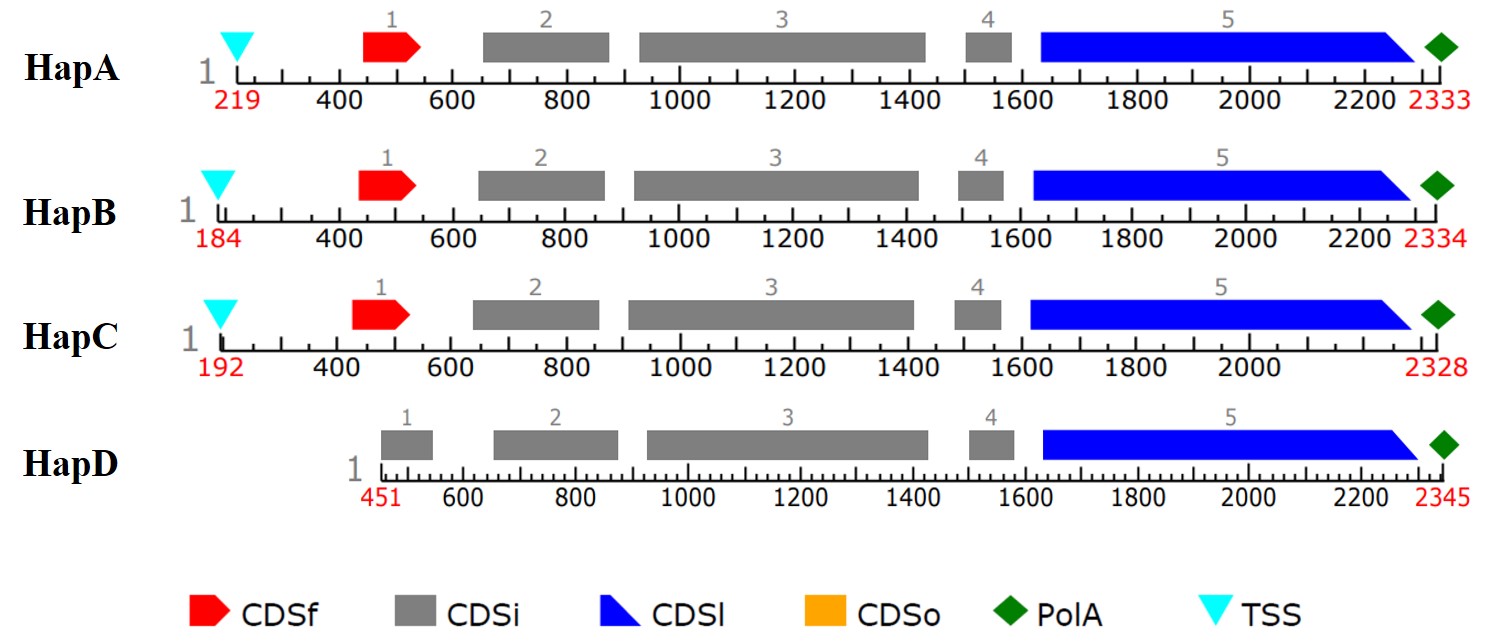


**Supplementary Fig. 1 Prediction of the four haplotypes of *SBM1* using website FGENESH** (http://linux1.softberry.com/berry.phtml?topic=fgenesh&group=programs&subgroup=gfind)
